# The effect of hormonal estrus induction on maternal effect and apoptosis-related genes expression in porcine cumulus-oocyte complexes

**DOI:** 10.1186/1477-7827-12-32

**Published:** 2014-05-01

**Authors:** Marek Bogacki, Marta Wasielak, Anna Kitewska, Iwona Bogacka, Beenu Moza Jalali

**Affiliations:** 1 Institute of Animal Reproduction and Food Research of Polish Academy of Sciences, Tuwima 10, Olsztyn 10-748, Poland; 2 Department of Animal Physiology, University of Warmia and Mazury, Oczapowskiego 2, Olsztyn 10-719, Poland

**Keywords:** Maternal effect genes, Pig, Estrus induction, Gonadotropins

## Abstract

**Background:**

The effect of hormonal estrus induction on maternal effect (*MATER* - maternal antigen that embryo requires, *ZAR-1* - zygote arrest 1, and *BMP15* - bone morphogenetic protein 15) and apoptosis-related genes expression (*BCL-2* and *BAX*) in porcine cumulus-oocyte complexes (COCs) and selected follicular parameters was investigated in this study.

**Methods:**

Gilts were divided into three groups: (I) with natural estrus; (II) stimulated with PMSG/hCG; and (III) with PMSG/hCG + PGF2alpha. Analysis of maternal effect and apoptosis-related transcripts expression in COCs, and progesterone synthesis pathway genes expression (*P450scc* and *3betaHSD)* in granulosa cells was performed by qPCR. BMP15 protein expression in follicular fluid (FF) was analyzed by western blot. Oocyte nuclear maturation was assessed by aceto-orcein staining. Progesterone (P4) and estradiol (E2) concentrations in FF and serum were measured by ELISA. Data were analyzed with the one-way ANOVA and Bonferroni post-test or Kruskal-Wallis test and Dunns post-test.

**Results:**

The highest expression of *MATER, ZAR-1*, and *BMP15* genes was found in COCs recovered from gilts treated with PMSG/hCG when compared to PMSG/hCG + PGF2alpha-stimulated or non-stimulated gilts. Hormonal treatment did not affect the BMP15 protein expression in FF, but increased the expression of genes participating in P4 synthesis in granulosa cells. The higher percentage of immature oocytes was found in PMSG/hCG-treated when compared to the non-stimulated gilts. The expression of *BCL-2* and *BAX mRNA,* and *BCL-2/BAX* mRNA ratio was significantly higher in COCs derived from PMSG/hCG-treated when compared to PMSG/hCG + PGF2alpha-treated or non-stimulated subjects. The level of P4 in serum was similar in animals from all experimental groups, while its concentration in FF was greater in gilts subjected to PMSG/hCG treatment than in PMSG/hCG + PGF2alpha-stimulated and non-stimulated gilts. The concentration of E2 did not differ in the serum or FF between the control group and the hormonally stimulated groups.

**Conclusions:**

Hormonal induction of estrus affected maternal effect gene transcripts levels in COCs and and oocyte nuclear maturation. The inclusion of PGF2alpha into the stimulation protocol enabled maintaining of physiological concentration of P4 in FF. Additionally, both hormonal treatments seem to be beneficial for apoptosis prevention through increasing *BCL-2/BAX* transcript ratio.

## Background

Hormonal induction of the estrus is commonly used in commercial swine breeding to improve production efficiency and facilitate animal management. It also serves as a fundamental technique in the preparation of recipients and donors for embryo transfer procedures, as well as in the recovery of a large number of embryos used later for both biotechnological purposes and in basic studies. However, the administration of gonadotropins may be to some extent detrimental to the reproductive performance of the treated animals. Estrus induction in gilts has an impact on follicular development, as reflected by the changes in follicle diameter and follicular fluid volume compared to naturally cyclic pigs [1]. In our earlier studies, a higher number of degenerated porcine embryos and a lower number of blastocysts hatched *in vitro* were found after hormonal induction of the estrus [2]. Moreover, studies have shown that hormonal treatment affects the expression of genes crucial for pre-implantation events in the uterus and conceptus during early pregnancy [3] and increases embryonic losses in pigs [4].

One of the predominant factors affecting proper embryo development and successful pregnancy is oocyte quality. During cytoplasmic maturation, the oocyte increases its size and volume, and a large number of mRNAs and proteins are accumulated at that time. These molecules, identified as maternal effect genes products, regulate events crucial for oocyte meiosis completion (nuclear maturation), organization of two pronuclei, and first embryo cleavages [5,6]. An expression of *ZAR-1* (zygote arrest 1), one of the maternal effect genes, was found in porcine oocytes and early embryos recovered *in vivo;* but it decreased significantly at the morula and blastocyst stages [7]. These indicate that *ZAR-1* has a significant function in oocyte development and during the first embryonic cleavages. *MATER* (maternal antigen that an embryo requires or *NALP5*), which also belongs to the maternal effect genes, was found to play a role in activating embryonic genome and maintaining chromosome stability and euploidy in mice [8,9]. Interestingly, recent data show that, during folliculogenesis, *MATER* is expressed and exerts its biological role also in the surrounding cumulus cells [10], which confirms the importance of the intimate relationship between oocyte and cumulus cells for the production of a fully competent gamete.

An example of another kind of maternal effect gene is bone morphogenetic protein 15 (*BMP15*). *BMP15* belongs to the TGF-β superfamily and is known as a granulosa cell mitosis and proliferation inducer [11,12]. The production of adequate amounts of BMP15 protein by the oocyte is necessary to promote cumulus cells expansion [13]. The expression of *BMP15* mRNA was also found in cumulus cells and it decreased along with the oocyte maturation and cumulus expansion *in vitro*[14]. Knockout of *BMP15* in female mice decreased fertility and diminished embryonic development [15]. There are studies demonstrating that the BMP15 level in follicular fluid (FF) appears to be a potential marker in predicting oocyte quality and subsequent embryo development [15,16].

Although there are some reports on maternal effect genes expression and their cellular localization during porcine oocyte maturation and embryo development *in vitro*[5,7,17-19], there is no evidence showing the effect of hormonal treatment on their expression in this species. The most commonly applied protocols of estrus induction include the use of a combination of PMSG to stimulate follicular development and hCG to induce ovulation. However, it was suggested by Sommer et al. [20] that including PGF2α into the PMSG/hCG protocol was very effective for collecting pronuclear stage embryos. Taking the following into consideration: (1) our previous results that estrus induction decreased embryo quality in pigs, (2) the information that genetic material within the oocyte plays an important role in directing the numerous events required for successful folliculogenesis and early embryo development, and (3) the fact that an luteinizing hormone (LH) surge causes the change from estradiol (E_2_) to domination of progesterone (P_4_), we investigated the effect of the two mentioned regimens of exogenous gonadotropins treatment on selected maternal effect gene expression (*MATER, ZAR-1* and *BMP15*) in COCs, steroid hormones concentration in FF, and P_4_ synthesis pathway gene expression (*P450scc* and *3βHSD*) in granulosa cells. Additionally, we examined apoptosis-related gene expression (antiapoptotic *BCL-2* and proapoptotic *BAX*) and *BCL-2/BAX* mRNA ratios in COCs in response to hormonal induction of the estrus.

## Methods

### Animals

The experiment was performed on gilts from one commercial herd, each 6.5 months old and on average weighing 115 kg. Gilts were divided into three groups (five to eight animals per group). Briefly, from the group of animals, the gilts entering into their natural estrus cycle were assigned to the natural cyclic group (group I), and the remaining gilts were assigned to groups II and III and treated hormonally to induce first and second estrus. Animals from group I (n = 8), exhibiting first and second estrus naturally, were not hormonally stimulated. Gilts were considered to be in estrus when they responded to boar exposure. The animals assigned to groups II and III were treated with PMSG [Folligon®; Intervet, Netherlands; 750 IU im], followed by hCG [Chorulon®; Intervet, Netherlands; 500 IU im] 72 h later. After seventeen days, the second estrus in animals from group II (n = 5) was induced by an identical treatment of PMSG and hCG. Animals from group III (n = 7), between days twelve and sixteen of the second estrus, were treated with PGF2α [Dinolitic®; Pfizer, Poland; 10 mg im], followed 24 h later with 10 mg of PGF2α simultaneously with 750 IU of PMSG, then followed 72 h later with 500 IU of hCG. This procedure was a modification of the method described previously by Sommer et al. [20]. The gilts were slaughtered about 36 h after hCG administration. Blood samples were collected, incubated overnight at 4°C and then centrifuged 3000 X g for 20 min at 4°C. Serum was harvested and frozen at -20°C for P_4_ and E_2_ analysis.

All procedures were conducted in accordance with the national guidelines for agricultural animal care and were approved by the Local Animal Ethics Committee, University of Warmia and Mazury in Olsztyn, Poland.

### Recovery of COCs and granulosa cells

The number of preovulatory follicles on each ovary was counted. Cumulus oocyte complexes (COCs) were recovered by cutting the follicles with a scalpel on a Petri dish. They were washed twice in Medium 199 (Sigma, St. Louis, MO, USA) supplemented with 0.68 mM L-glutamine (Sigma), 20 mM Hepes (Sigma), 100 U/ml penicillin (Sigma), 0.1 mg/ml streptomycin (Sigma) and 10% fetal bovine serum (FBS; Invitrogen, Carlsbad, CA, USA). After washing twice in phosphate-buffered saline, the COCs from one animal were pooled in groups of ten to fifteen per tube, snap frozen in liquid nitrogen, and kept at -80°C until further RNA isolation. At the same time, FF was collected (per animal – FF from all preovulatory follicles from both ovaries was pooled), centrifuged to remove cell debris, and frozen at -20°C for P_4_ and E_2_ analysis. The granulosa cells were gently scraped from the inner follicle wall and transferred to a 1.5 ml microcentrifuge tube in phosphate-buffered saline and centrifuged at 90 X g for 10 min at room temperature. After centrifugation, the supernatant was removed and the cells were snap frozen in liquid nitrogen and stored at -80°C for further RNA isolation.

### Evaluation of nuclear maturation

For evaluation of oocyte nuclear maturation, additional animals prepared as described previously were used [natural estrus (group I) n = 4; PMSG/hCG (group II) n = 3; PMSG/hCG + PGF2α (group III) n = 3]. After the recovery of COCs, cumulus cells were removed completely by vortexing in 0.1% (w/v) hyaluronidase (Sigma). Denuded oocytes were placed on a glass slide under a cover slip (supported with Vaseline corners) and fixed for up to 72 h in an acetic acid/ethanol fixative (1:3, v:v). Nuclear structures were then visualized by staining with aceto-orcein (1% orcein in 45% acetic acid). Oocytes were evaluated under a phase-contrast microscope for the stage of nuclear maturation. Based on the stage of nuclear maturation, oocytes were divided into two groups: immature oocytes (GV) and oocytes that resumed meiosis (prometaphase I – metaphase II).

### P_4_ and E_2_ measurement

Concentrations of P_4_ and E_2_ in FF and serum were determined using commercial ELISA kits (Enzo Life Sciences, New York, NY, USA) according to the supplier’s instructions. The standard curves for P_4_ and E_2_ ranged from 15.6 to 500 pg/ml and from 15.6 to 1000 pg/ml, respectively. The concentrations of P_4_ and E_2_ in FF and serum were measured in duplicates for each sample and the final concentration was expressed as an average. Assay sensitivity was 8.57 pg/ml for P_4_ and 14.00 pg/ml for E_2_. The intra-assay coefficients of variation were 5.9% for P_4_ and 4.9% for E_2_.

### RNA isolation and qPCR

Total RNA from COCs and granulosa cells was isolated using Qiagen RNeasy® Plus Micro Kit (Qiagen, Valencia, CA, USA) and checked for quantity and quality with NanoDrop® (ND-1000; Thermo Scientific, Waltham, MA, USA). DNase treatment with gDNA eliminator columns (Qiagen) was included in the RNA isolation protocol. The RNA obtained was reversely transcribed with the use of a transcriptor high fidelity cDNA synthesis kit (Roche, Basel, Switzerland). For reverse transcription, 15 ng of total RNA from COCs and 1 μg of RNA from granulosa cells were used. The reaction was performed in a total volume of 20 μl, including RNA, water, 60 μM of random hexamer primers, reaction buffer, 5 mM DTT, 20 U Protector RNase inhibitor, 1 mM deoxynucleotide mix, and 10 U of reverse transcriptase. At first, the template-primer mixture was denatured by heating the tube for 10 min at 65°C in a thermo cycler (SensoQuest GmbH, Göttingen, Germany). Then, after adding the remaining components of the mixture, the following thermal profile of the reaction was applied: 30 min at 45°C followed by inactivation of reverse transcriptase at 85°C for 5 min, with subsequent cooling to 4°C. cDNA was kept at -20°C for further qPCR analysis.

Power SYBR Green PCR Master Mix (Life Technologies, Carlsbad, CA, USA) was used for qPCR analysis. The primers used for qPCR, products sizes, and GenBank accession numbers and/or references are included in Table 1. The qPCR mix consisted of 2 μl of RT product, 1 μl of forward and reverse primer (0.4 μM), 8.5 μl of nuclease-free water, and 12.5 μl of SYBR Green. The reaction was performed manually in duplicates for each sample, at a final volume of 25 μl in 96-well plates using ABI 7300 (Life Technologies). Each run included a non-template control (NTC). A standard curve was generated by amplifying serial dilutions of a known quantity of cDNA. The amplification efficiency for each gene was found to be between 90 and 100% for all the investigated genes. The thermal profile for amplification of the investigated genes was as follows: preincubation at 95°C for 15 min, followed by 45 cycles of denaturation at 95°C for 15 s, annealing at either 52°C (for glyceraldehyde-3-phosphate dehydrogenase; *GAPDH*), 55°C (for *BMP15, MATER, ZAR-1*), 57°C (for *BCL-2*), or 60°C (for *BAX, P450scc, 3βHSD*) for 30 s, and elongation at 72°C for 30 s. After the end of the last cycle, the melting curve was generated. Product purity was confirmed by electrophoresis and its specificity was confirmed by sequencing (Genomed, Warsaw, Poland). The obtained sequences were compared with the expected sequences of the investigated genes using BLAST (bl2seq). The final quantification was reported as a relative expression (average value from duplicates) after normalization to reference gene (*GAPDH*) expression in the same samples. *GAPDH* was selected as a good reference gene candidate for pig oocytes and embryos for the reasons suggested by Kuijk et al. [21]. There was no statistically significant impact of the treatments on *GAPDH* transcript level in our study, confirming its usefulness as a good endogenous control.

**Table 1:** Primers used for qPCR

**Gene**	**Primer sequence**	**Amplicon size (base pairs)**	**GenBank accession no./reference**
*MATER*	F: GATTAACGCCCAGCTCTTGT	154	AM748274.1
	R: AGCTTCTGCAGAGTGCAGTG		
*ZAR-1*	F: TGGTGTGTCCAGGGCACTAA	213	NM_001129956
	R: GTCACAGGAGAGGCGTTTGC		
*BMP15*	F: AGCTTCCACCAACTGGGTTGG	285	Li et al. [17]
	R: TCATCTGCATGTACAGGGCTG		
*BAX*	F: AAGCGCATTGGAGATGAACT	159	Wasielak et al. [22]
	R: AAAGTAGAAAAGCGCGACCA		
*BCL-2*	F: GAAACCCCTAGTGCCATCAA	196	Ju et al. [23]
	R: GGGACGTCAGGTCACTGAAT		
*P450scc*	F: TTTACAGGGAGAAGCTCGGCAAC	251	Walzel et al. [24]
	R: TTACCTCCGTGTTCAGGACCAAC		
*3βHSD*	F: GGGTTTCTGGGTCAGAGGATC	236	Walzel et al. [24]
	R: CGTTGACCACGTCGATGATAGAG		
*GAPDH*	F: TCGGAGTGAACGGATTTG	219	Kuijk et al. [21]
	R: CCTGGAAGATGGTGATGG		

### Western blot

A western blot of the BMP15 protein was carried out according to the method described previously by Wu et al. [15] and Paradis et al. [25]. Protein concentration in FF was determined according to the Bradford [26]. Briefly, 0.75 μg of protein in an SDS-gel loading buffer (50 mM TRIS-HCl, 4% SDS, 20% glycerol, and 2% β-mercaptoethanol) was heated to 95°C for 4 min, electrophoretically separated on a 12% polyacrylamide-SDS gel for 1.5 h at a constant current (200 mA), and then transferred overnight onto a nitrocellulose membrane. After the transfer, the membranes were stained with Ponceau S for total protein loading. They were then blocked in a solution of 5% (w/v) non-fat dry milk for 1.5 h. The expression of BMP15 was determined with the use of a primary polyclonal rabbit anti-human BMP15 antibody (Santa Cruz Biotechnology, Inc., Santa Cruz, CA, USA), diluted 1:400 in blocking solution. The secondary antibody used was goat anti-rabbit IgG conjugated with alkaline phosphatase (Sigma), diluted 1:20,000 in blocking solution containing pig protein. The molecular weight of the bands was determined by reference to a standard molecular weight marker. Three immunoreactive bands were found, representing BMP15 promature protein (65 kDa), cleaved proregion (50 kDa), and mature protein (25 kDa). The intensity of the bands was quantified by measuring optical density using Kodak® ID image analysis software (Eastman Kodak, Rochester, NY, USA). A control sample (a mix of all samples analyzed) was loaded on each gel to correct for interblot variability [15,25]. Densitometric values for individual FF samples were normalized to a stable protein band quantified after Ponceau S staining. All samples were electrophoresed and analyzed in duplicate, and values were averaged before statistical analysis [15,25].

### Statistical analysis

The statistical analysis was carried out with the use of GraphPad PRISM v. 5.0 software (GraphPad Software, Inc., San Diego, CA, USA). Normality (bell shaped distribution) and homoscedasticity (variances) of the data were tested before analysis. For the data fitting the assumptions of parametric tests, i.e., following normal distribution and having similar variances, a one-way ANOVA and Bonferroni post-test was applied. For data that did not meet the assumptions of parametric tests (not following normal distribution or having different variances), the non-parametric Kruskal-Wallis test and Dunn post-test were done.

With regard to the pigs’ reproductive parameters (such as the number of follicles, oocyte nuclear maturation, hormone concentration in FF and serum), the data did not show normal distribution and/or similar variances even after logarithmic or arcsine transformation (in case of the data concerning oocyte nuclear maturation); therefore we analyzed them with the non-parametric Kruskal-Wallis test and Dunn post-test. Because of different variances, the data of *ZAR-1, BMP15, BAX*, and *BCL-2/BAX* mRNA expression were logarithmically transformed and analysis was done on the transformed data. These data were analyzed with the use of a one-way ANOVA and Bonferroni post-test. *MATER, BCL-2, P450scc*, *3βHSD* mRNA and BMP15 protein expression data, which did not show normal distribution, were analyzed with the use of the non-parametric Kruskal-Wallis test and Dunn post-test. P values less than 0.05 were considered significant. Results are presented as ls mean ± S.E.M.

## Results

### The effect of hormonal treatments on follicle numbers, oocyte nuclear maturation, and steroids concentrations (P_4_ and E_2_) in FF and blood serum

**Figure 1:**
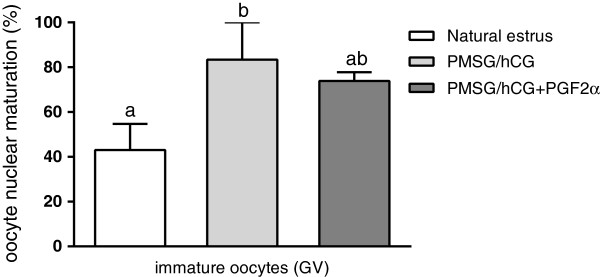
**The nuclear maturation of oocytes.** The comparison of nuclear maturation of oocytes derived from non-stimulated gilts with natural estrus (group I; n = 4), stimulated with PMSG/hCG (group II, n = 3) or with PMSG/hCG + PGF2α (group III, n = 3). Values are expressed as percentages of immature oocytes (germinal vesicle; GV). Significant differences between groups were marked with p < 0.05.

The number of preovulatory follicles per gilt did not differ between the experimental groups of gilts (Table 2). A higher percentage of immature oocytes was found in PMSG/hCG-treated animals (group II) when compared to the naturally cyclic animals (group I) (p < 0.05; Figure 1). The percentage of oocyte nuclear maturation did not differ between PMSG/hCG-treated animals (group II) and PMSG/hCG + PGF2α-treated animals (group III) (p > 0.05).

**Table 2:** The effect of hormonal treatments on follicle numbers and steroids concentrations in FF and serum

	**Treatment**			**p**
	**Natural estrus**	**PMSG/hCG**	**PMSG/hCG + PGF2α**	
*Follicles per gilt*	20.13 ± 2.33	14.00 ± 2.14	15.43 ± 1.87	ns
***Steroids in FF***				
*P*_*4*_*(ng/ml)*	50.24 ± 7.47^a^	223.2 ± 31.38^b^	80.03 ± 11.03^a^	a vs. b
				p < 0.01
*E*_*2*_*(ng/ml)*	13.18 ± 4.35	6.176 ± 2.25	5.50 ± 1.12	ns
***Steroids in blood serum***				
*P*_*4*_*(ng/ml)*	1.86 ± 0.21	1.25 ± 0.16	1.28 ± 0.16	ns
*E*_*2*_*(pg/ml)*	55.24 ± 9.18	47.45 ± 5.41	71.95 ± 21.08	ns

Follicles number and P_4_ and E_2_ concentration in follicular fluid and blood serum recovered from non-stimulated gilts with natural estrus (group I; n = 8), stimulated with PMSG/hCG (group II; n = 5) or with PMSG/hCG + PGF2α (group III; n = 7). Values are expressed as ls mean ± S.E.M.

P_4_ concentration in blood serum did not differ between the experimental groups of animals (p > 0.05; Table 2). However, its concentration in FF was significantly higher in gilts stimulated with PMSG/hCG (group II) compared to those stimulated with PMSG/hCG + PGF2α (group III) or in the naturally cyclic animals (group I) (p < 0.01; Table 2). There was no difference in P_4_ concentration in FF between naturally cyclic (group I) and PMSG/hCG + PGF2α-treated animals (group III) (p > 0.05).

There was no difference in E_2_ concentration in blood serum between the PMSG/hCG-treated gilts (group II), PMSG/hCG + PGF2α-treated gilts (group III), and naturally cyclic gilts (group I) (p > 0.05; Table 2). The same observation was made regarding E_2_ concentration in FF (Table 2).

### The effect of hormonal treatments on the expression of maternal effect (*MATER, ZAR-1, BMP15*) and apoptosis-related (*BCL-2* and *BAX*) genes, *BCL-2/BAX* mRNA ratio in COCs and BMP15 protein in FF

**Figure 2:**
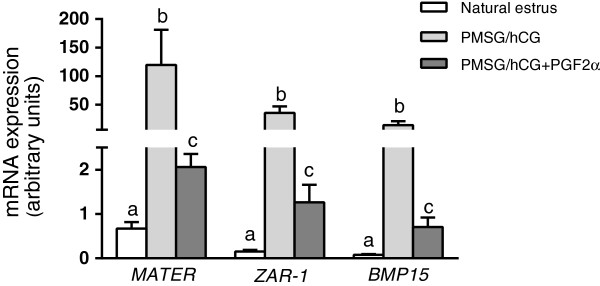
**The expression of maternal effect genes in porcine COCs.** The comparison of the expression of maternal effect genes (*MATER, ZAR-1* and *BMP15*) in COCs derived from non-stimulated gilts with natural estrus (group I; n = 8), stimulated with PMSG/hCG (group II; n = 5) or with PMSG/hCG + PGF2α (group III; n = 7). Values from qPCR were normalized to *GAPDH* expression. Different superscripts depict significant statistical differences at p < 0.05. Data are expressed in arbitrary units as the ls mean ± S.E.M.

The highest expression of *MATER, ZAR-1, BMP15* genes was found in the COCs recovered from gilts treated with PMSG/hCG (group II), when compared to the PMSG/hCG + PGF2α-stimulated (group III) and naturally cyclic gilts (group I) (p < 0.01; Figure 2). Additionally, maternal effect gene expression was higher in the COCs of PMSG/hCG + PGF2α-treated gilts (group III) than in the naturally cyclic gilts (group I) (p < 0.05).

**Figure 3:**
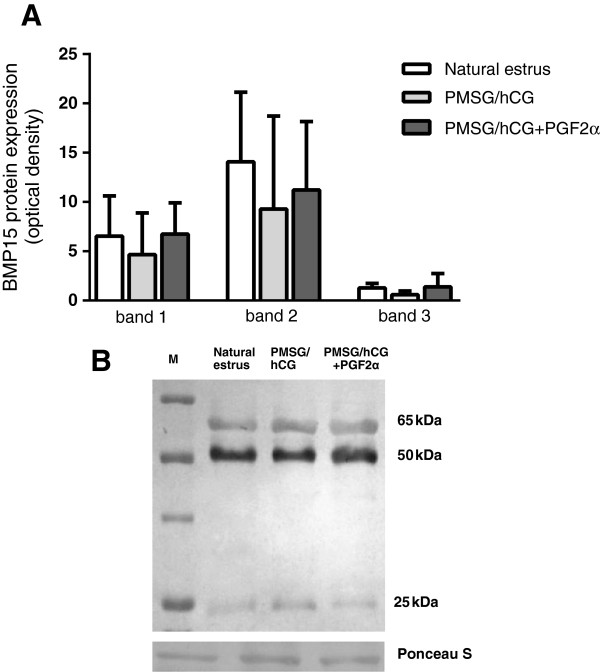
**The expression of BMP15 protein in FF.** The comparison of expression of BMP15 protein **(A)** in follicular fluid derived from non-stimulated gilts with natural estrus (group I; n = 8), stimulated with PMSG/hCG (group II; n = 5) or with PMSG/hCG + PGF2α (group III; n = 7). Representative bands of western blots are presented **(B)**. The bands represent three forms of BMP15 found in FF: premature protein (band 1, ~65 kDa), cleaved proregion (band 2, ~50 kDa) and mature protein (band 3, ~25 kDa). Data are expressed in arbitrary optical density units as the ls mean ± S.E.M. Different superscripts depict significant statistical differences at p < 0.05.

Hormonal treatment did not affect BMP15 protein level in FF. The densitometric analysis of all three detected bands of BMP15 (analyzed separately) indicated that there were no differences in BMP15 forms expression in FF between experimental groups (marked at p < 0.05; Figure 3).

**Figure 4:**
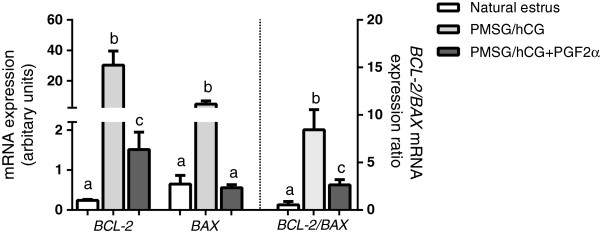
**The expression of apoptosis-related genes in porcine COCs.** The comparison of the expression of apoptosis-related genes *BCL-2, BAX* and *BCL-2/BAX* mRNA ratio in COCs derived from non-stimulated gilts with natural estrus (group I; n = 8), stimulated with PMSG/hCG (group II; n = 5) or with PMSG/hCG + PGF2α (group III; n = 7). Values from qPCR were normalized to *GAPDH* gene expression. Data are expressed in arbitrary units as the ls mean ± S.E.M. Different superscripts depict significant statistical differences at p < 0.05.

The expression of apoptosis-related gene *BCL-2* was significantly higher (p < 0.05) in COCs derived from gilts treated with PMSG/hCG (group II; Figure 4), compared to PMSG/hCG + PGF2α-treated gilts (group III) or the naturally cyclic animals (p < 0.05); Figure 4. Moreover, its expression was enhanced in COCs recovered from PMSG/hCG-treated gilts (group II) compared to PMSG/hCG + PGF2α-treated gilts (group III) (p < 0.05).

In the case of *BAX* mRNA expression, it was significantly higher in COCs of PMSG/hCG-stimulated gilts (group II) compared to PMSG/hCG + PGF2α-stimulated gilts (group III) and the naturally cyclic gilts (group I) (p < 0.05; Figure 4). The expression of *BAX* mRNA was comparable in the PMSG/hCG + PGF2α-treated gilts (group III) and in the naturally cyclic gilts (group I) (p > 0.05).

The ratio of *BCL2/BAX* mRNA was higher in COCs of gilts treated with PMSG/hCG (group II) than in the other two groups (p < 0.01; Figure 4). Additionally, a significantly higher *BCL-2/BAX* mRNA ratio was found in PMSG/hCG + PGF2α-treated (group III) animals than in animals attaining estrus naturally (group I) (p < 0.05).

### The effect of hormonal treatments on the expression of P_4_ synthesis pathway genes (*P450scc and 3βHSD*) in granulosa cells

**Figure 5:**
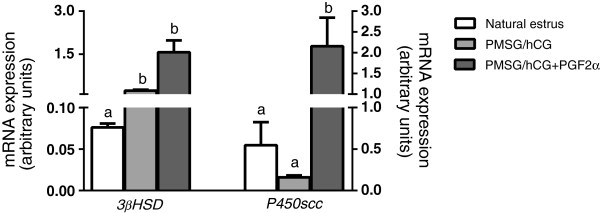
**The expression of P**_**4 **_**synthesis pathway genes in porcine granulosa cells.** The comparison of the expression of P_4_ synthesis pathway genes (*3βHSD* and *P450scc*) in granulosa cells derived from non-stimulated gilts with natural estrus (group I; n = 8), stimulated with PMSG/hCG (group II; n = 5) or with PMSG/hCG + PGF2α (group III; n = 7). Values from qPCR were normalized to *GAPDH* expression. Different superscripts depict significant statistical differences at p < 0.05. Data are expressed in arbitrary units as the ls mean ± S.E.M.

Regarding expression of *P450scc* mRNA, it was significantly increased in granulosa cells from females subjected to hormonal treatment with PMSG/hCG + PGF2α (group III) in comparison to granulosa cells recovered from naturally cyclic animals (group I) and PMSG/hCG-treated animals (group II) (p < 0.05; Figure 5). Additionally, we did not find any difference in *P450scc* mRNA expression in granulosa cells obtained from gilts stimulated with PMSG/hCG (group II) and the naturally cyclic gilts (group I) (p > 0.05; Figure 5).

The expression of *3βHSD* mRNA was significantly increased in granulosa cells recovered from gilts stimulated with PMSG/hCG + PGF2α (group III), compared to its expression in granulosa cells obtained from naturally cyclic gilts (group I) (p < 0.001; Figure 5). Moreover, its expression was also increased in granulosa cells of PMSG/hCG-treated animals (group II) compared to naturally cyclic pigs (group I) (p < 0.05).

## Discussion

The paper presents the results on the effect of hormonal estrus induction on COCs quality and selected follicular parameters in the pig.

We found that hormonal stimulation of gilts with PMSG/hCG or PMSG/hCG + PGF2α did not affect the follicle number when compared with naturally cyclic animals. It had been previously reported that in pregnant females the number of corpora lutea and the fertilization rate were comparable between non-stimulated and PMSG/hCG-treated pigs [3]. On the other hand, Kiewisz et al. [27] showed that treatment with PMSG/hCG + PGF2α generated a higher number of corpora lutea and conceptuses when compared to animals attaining estrus naturally. This observation was not confirmed in the present study with regard to follicular growth; however, the discrepancy may result from differences in animal treatment. In the cited study of Kiewisz et al. [27], gilts were assigned for hormonal stimulation in their third estrus, while in our study animals with two consecutively stimulated estruses were used.

In the present study, we observed a markedly higher expression of all the investigated maternal effect gene transcripts (*MATER, ZAR-1, BMP15*) in COCs recovered from animals treated hormonally with PMSG/hCG or PMSG/hCG + PGF2α, when compared with those observed in naturally cyclic females. This high expression of maternal effect genes, particularly in COCs obtained from PMSG/hCG-stimulated pigs, seems to be associated with a higher number of immature oocytes that did not resume meiosis. It is well known that the intense synthesis of maternal transcripts precedes the onset of transcriptional silencing during the GV stage. The maternal transcripts undergo post-transcriptional regulation through shortening of the polyA tail, which enables their storage and protection from the translation machinery. After meiosis resumption, when the proteins are required, the transcripts are polyadenylated and used for translation, then rapidly degraded [18,28]. The high levels of the investigated maternal effect gene transcripts found in our study in COCs from hormonally treated animals, and the lower percentage of oocytes that resumed meiosis, may result from the fact that cellular mechanisms controlling transcription and/or translation in female gametes can be modified in these gilts in response to exogenous gonadotropins. Similarly, in studies on the development of porcine embryos, it was shown that disturbation in maternal mRNA degradation may be responsible for delayed cleavage of porcine embryos *in vitro*[29]. Together, these findings suggest that mechanisms of maternal transcript utilization are potential determinants of oocyte/embryo quality and developmental potential, and may be affected by factors such as hormonal stimulation and *in vitro* culture conditions.

The BMP15 protein level in FF is considered as potential marker of oocyte quality. The main role played by BMP15 protein in preovulatory follicles is to stimulate cumulus cell expansion [13,14]. The present study has shown that hormonal treatment of gilts markedly increased *BMP15* transcript level in COCs but it did not affect BMP15 protein level in FF. Similar results were obtained by Paradis et al. [25] who also did not find correlation between *BMP15* mRNA expression in oocytes and protein in FF during different phases of follicle development, suggesting that not necessarily protein by itself but rather its receptor can be influenced by gonadotropins in the pig.

In our experiment, the serum concentration of P_4_ did not differ between the experimental groups but was higher in the FF of PMSG/hCG compared to PMSG/hCG + PGF2α-stimulated and non-stimulated females. Similarly, in the studies of Wiesak et al. [1] the concentration of P_4_ in FF, recovered from follicles of non-stimulated gilts, was lower than in follicles obtained from gonadotropin-treated (PMSG/hCG) gilts at all stages of follicle development. The high concentration of P_4_ in FF obtained from PMSG/hCG-treated animals was associated with increased percentage of oocytes at the GV stage and increased levels of maternal effect gene transcripts in COCs. Shimada and Terada [30] found that P_4_ induces germinal vesicle breakdown (GVBD) in porcine oocytes, probably through the disruption of gap junction communication. Dynamic changes in the expression of P_4_ receptor proteins, following *in vitro* maturation in response to supplementation with LH, follicle-stimulating hormone (FSH), or P_4,_ also indicated the role of P_4_ in bovine oocyte maturation [31]. In the current *in vivo* study, the increased concentration of P_4_ in FF of PMSG/hCG-treated animals does not seem to support meiosis resumption in the oocytes. On the other hand, it was found that the addition of various concentrations of P_4_ to the maturation medium had negative effects on the percentage of oocytes completing nuclear or cytoplasmic maturation in porcine [32] and bovine oocytes [33].

Our results also indicate that the inclusion of PGF2α into the synchronization protocol decreases the P_4_ concentration in follicles to the levels observed in the follicles of naturally cyclic gilts. A similar effect was observed in *in vitro* studies, where PGF2α exerted a concentration-dependent inhibitory influence on gonadotropin-stimulated P_4_ production [34]. It can be suggested that including PGF2α into an estrus stimulation protocol in the pig can be beneficial for obtaining more physiological concentration of P_4_ in FF and enhancing the natural hormonal environment for oocyte development. Maintaining the appropriate P_4_ concentration in FF is a possible explanation for the results of Sommer et al. [20], in which inclusion of PGF2α into gonadotropin stimulation protocol was very effective for the collection of pronuclear stage embryos for biotechnological purposes.

From the present study it seems that hormonal stimulation of estrus increases mRNA expression of both investigated enzymes participating in P_4_ synthesis (*P450scc* and *3βHSD*) in granulosa cells. While in the granulosa cells of PMSG/hCG-treated animals only *3βHSD* expression was raised, adding PGF2α into the stimulation protocol enhanced the expression of both investigated steroidogenesis-related genes. Similarly to our findings, the studies of Blitek et al. [35] found that induction of estrus with PMSG/hCG + PGF2α increased the expression of mRNA for steroidogenic pathway genes such as *StAR*, *CYP11A*, and *3βHSD* in the corpus luteum on days 9 and 12 of pregnancy, compared to the animals attaining estrus naturally.

Interestingly, in our study the increase of *3βHSD* mRNA expression was observed in granulosa cells after stimulation with both PMSG/hCG and PMSG/hCG + PGF2α; however, the increased P_4_ concentration was found only in animals treated with PMSG/hCG. It may be that the inclusion of PGF2α in the stimulation protocol increased P_4_ metabolism, while at the same time not affecting *3βHSD* mRNA expression stimulated by gonadotropins. A slightly different situation was found in the case of *P450scc* mRNA, where we did not observe the effect of gonadotropins treatment on its expression; however, its enhanced expression is rather a result of the increased metabolism of P_4_ induced by PGF2α.

The intra-follicular concentration of E_2_ is considered as a marker for preovulatory maturation, and it increases progressively along with follicle development. In our experiments, both naturally cyclic and hormonally stimulated gilts had a similar E_2_ concentration in blood serum and FF. However, the results obtained by Wiesak et al. [1] revealed lower levels of E_2_ in the follicles of hormonally treated gilts compared to naturally cyclic animals. In the present study we did not find a relationship between E_2_ concentration and the resumption of meiosis in oocytes. This observation is in agreement with the findings of *in vitro* experiments [36], where it was suggested that E_2_ is not involved in nuclear maturation of pig oocytes but rather may promote changes in calcium activity during oocyte cytoplasmic maturation [37].

It has been suggested that oocytes of inferior quality are predestined to undergo apoptosis. Changes in the balance between anti- and proapoptotic factors play an important role in the regulation of cellular apoptosis. Our observation that gonadotropins increased the *BCL-2/BAX* transcript ratio in COCs is in agreement with the antiapoptotic effect on the whole follicles found by Chun et al. [38]. Moreover, it was revealed that PMSG/hCG may suppress apoptotic machinery in rodent granulosa cells [39,40]. In our study, a high level of P_4_ in FF was associated with an increased ratio of BCL-2/BAX mRNA in COCs of PMSG/hCG-stimulated animals. It is possible that this gonadotropin-induced antiapoptotic effect in COCs is mediated through a high P_4_ content in the FF. In bovine granulosa cells, gonadotropin surges induced the expression of progesterone receptors, promoting their resistance to apoptosis [41]. This relationship was also found in bovine cumulus cells, where treatment with P_4_ for 24 h decreased caspase-3 activity and the ratio of *BAX/BCL2* transcripts, while an inhibition of P_4_ synthesis enzymes increased caspase-3 activity and the apoptosis in these cells [42]. On the other hand, the ability of oocytes to prevent apoptosis may rely on some maternal specific factors, like BMP15 [43]. The role of BMP15 in apoptosis prevention might be suggested in our study, where high *BMP15* transcript levels were found in COCs recovered from hormonally treated animals.

## Conclusions

In conclusion, hormonal induction of estrus with PMSG/hCG or PMSG/hCG + PGF2α affected maternal effect gene transcript levels in porcine COCs and oocyte maturation. The high maternal effect genes mRNA content in COCs from hormonally stimulated animals may be caused by some disturbances in maternal transcripts utilization after gonadotropin treatment. The inclusion of PGF2α into the stimulation protocol seems to be beneficial for maintaining physiological concentration of P_4_ in FF. Moreover, both hormonal regimens appear to be beneficial for apoptosis prevention through increasing the *BCL-2/BAX* ratio. Whether the effect of gonadotropins on oocyte maturation and maternal effect gene expression affect also oocyte quality and subsequent embryo development needs to be further studied.

## Competing interests

The authors declare that they do not have competing interests.

## Authors’ contributions

MB conceived of the study, participated in its design, coordination and helped to draft the manuscript. MW participated in coordination, collected the tissues and carried out mRNA isolation, qPCR and aceto-orcein staining. AK collected the tissues and carried out Western blot analysis. IB participated in preparation of the manuscript draft and qPCR analysis. BMJ carried out immunoassays. All authors read and approved the final manuscript.
